# Genome Sequence of the Endosymbiont *Endozoicomonas* sp. Strain GU-1 (*Gammaproteobacteria*), Isolated from the Staghorn Coral Acropora pulchra (Cnidaria: Scleractinia)

**DOI:** 10.1128/mra.01355-22

**Published:** 2023-02-21

**Authors:** Pablo De la Vega, Gaurav G. Shimpi, Bastian Bentlage

**Affiliations:** a Marine Laboratory, University of Guam, Mangilao, Guam, USA; Montana State University

## Abstract

*Endozoicomonas* sp. strain GU-1 was isolated from two separate staghorn coral (Acropora pulchra) colonies collected in Guam, Micronesia. Both isolates were grown in marine broth prior to DNA extraction and Oxford Nanopore Technologies (ONT) sequencing. Genomes were approximately 6.1 Mbp in size, containing highly similar gene content and matching sets of rRNA sequences.

## ANNOUNCEMENT

*Endozoicomonas* spp. are endosymbiotic bacteria of marine animals (1) and dominant members of the coral microbiome (2), benefiting the metabolism of their host (3). The few *Endozoicomonas* genome sequences available to date contain an abundance of transposable elements which likely allow for quick adaptation to new hosts and environments (4). Here, we provide genome sequences from two isolates (Ap1-3 and Ap2-2) of a so-far undescribed *Endozoicomonas* strain, an endosymbiont of the coral Acropora pulchra. These genome sequences add new data that will facilitate studies of *Endozoicomonas* gene synteny and the potential association of transposable elements with genome rearrangements.

**FIG 1:**
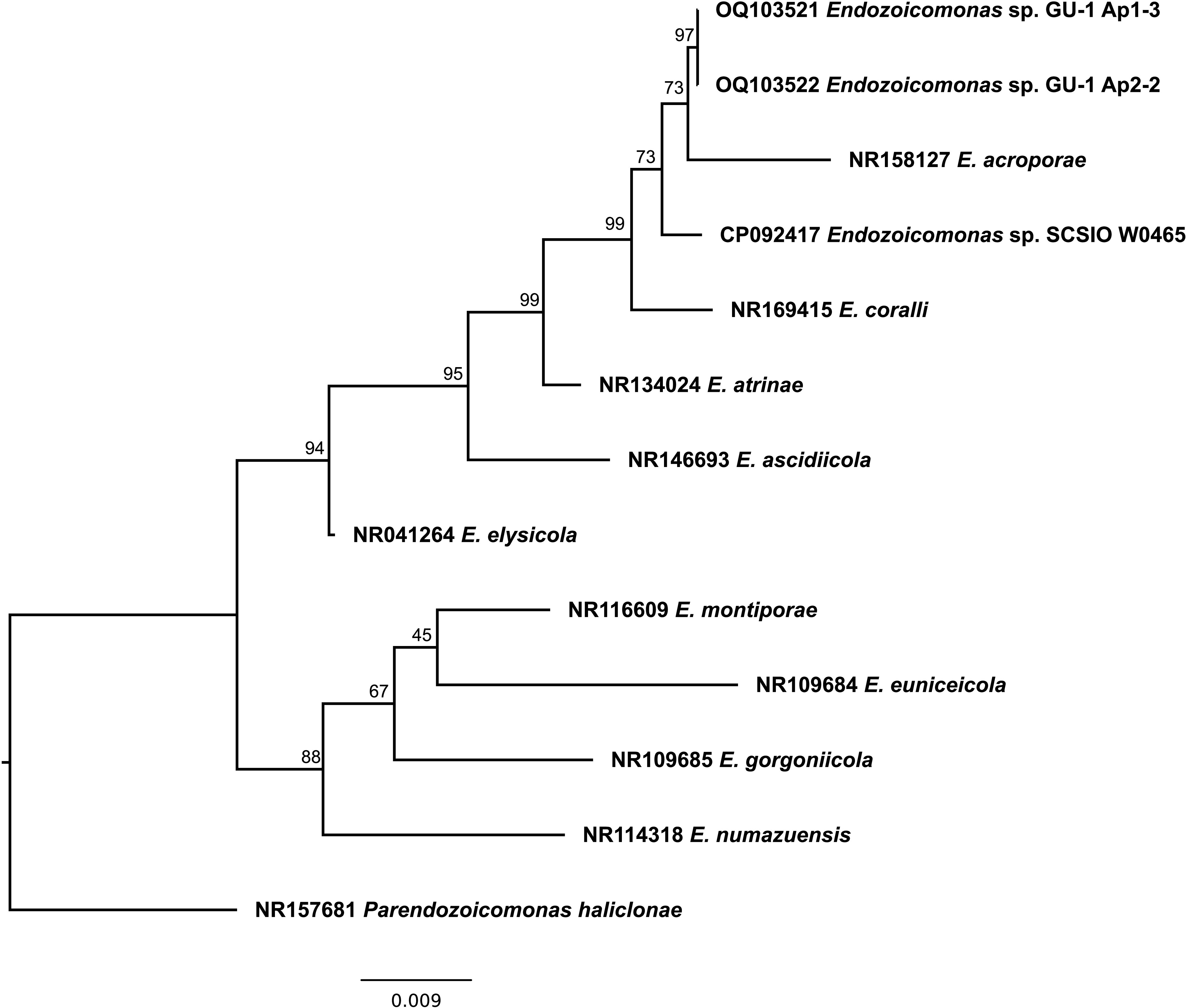
*Endozoicomonas* 16S maximum likelihood phylogeny inferred under the TIM3+I+G model. Bootstrap supports on nodes were calculated using 1,000 nonparametric replicates; scale bar indicates substitutions per site.

Roughly 5-cm-long *Acropora pulchra* fragments were collected from two separate coral colonies growing some 20 m apart in shallow (~1 m) water on the fringing Tanguisson reef flat in northwest Guam (13°32′56.4″N 144°48′36.0″E). Coral tissue was removed from the coral skeleton using an airbrush with sterile seawater and disrupted using a 2-mL dounce homogenizer. Next, 50-μL replicates of undiluted and 10-fold diluted tissue homogenate from each specimen were plated onto marine agar and incubated at 23°C for 4 days. A total of 5 mL of marine broth was inoculated with single colonies, and cultures were incubated for 48 h at 23°C. *Endozoicomonas* sp. strain GU-1 colonies were identified by DNA barcoding using an almost complete, 1,286-bp-long fragment of 16S amplified with rRNA primer pairs 27F/mEn771R and En667F/1492R (5–7) in conjunction with phylogenetic analysis using raxmlGUI 2.0 (8, 9) (Fig. 1).

Following DNA barcoding, isolates identified as *Endozoicomonas* sp. GU-1 were used to inoculate 5 mL of marine broth, as described above. High-molecular-weight (HMW) DNA was extracted from these cultures using the Monarch HMW DNA extraction kit T3060 (New England BioLabs, Ipswich, MA). Libraries were prepared using ligation sequencing (SQK-LSK112) and native barcoding kits (SQK-NBD112) to multiplex samples for Nanopore sequencing on a MinION instrument (Oxford Nanopore Technologies, Oxford, UK). Bases were called from raw data using Guppy v6.3.7 (Oxford Nanopore Technologies) followed by removal of adapters with Porechop v0.2.4 (10). Flye v2.9.1 (11) was used for initial assembly and Medaka v1.7.2 (Oxford Nanopore Technologies) for genome polishing. The completeness of genome assemblies was assessed using the BUSCO (12) *Oceanospirillales* gene set, followed by annotation with the NCBI Prokaryotic Genome Annotation Pipeline (PGAP) v6.4 (13). While the standard for ONT-based bacterial genome assemblies has been the incorporation of Illumina data for error correction (14), flow cell and sequencing chemistry upgrades released in early 2022, and employed by us, provide reads with error rates below 1%, improving assembly quality (15).

Assemblies for both isolates yielded a circular chromosome of almost 6.1 Mbp in size (Table 1), in addition to a combined total of four short, low coverage contigs ranging from ~2 kb to ~8 kb that were removed from final assembly files. Final assemblies contained almost 95% of the 619 single-copy *Oceanospirillales* BUSCO gene set (Table 1). A total of 24 rRNAs were identified (Table 1), which is comparable to the high rRNA operon copy numbers present in other *Endozoicomonas* genomes. The high rRNA copy number may be a trait linked to *Endozoicomonas*-host nutrient cycling, given the association of rRNA operon copy number with bacterial resource exploitation (16 and 17) and reproduction (18). Interest in *Endozoicomonas* genome evolution and function has grown in recent years due to its central role in coral holobiont homeostasis and health (19). The genome sequences disseminated here add to the resources available for understanding *Endozoicomonas*-coral interactions.

**TABLE 1:** *Endozoicomonas* sp. GU-1 genome assembly summary statistics

NCBI accession no.	Genome size (bp)	Coverage (×)	ONT read no.	Read *N*_50_ (bp)	GC content (%)	No. of^*a*^:				Genome completeness^*b*^ (%)
						CDSs	23S	16S	5S	
CP114886	6,098,955	263	103,652	29,955	49.5	5,014	7	7	8	94.5
CP114771	6,085,864	159	106,878	20,499	49.5	4,956	7	7	8	94.0

a The number of coding sequences (CDSs) and rRNAs (23S, 16S, and 5S) were predicted using PGAP.

b Genome completeness was assessed using the set of 619 *Oceanospirillales* genes provided by BUSCO.

### Data availability.

ONT reads were deposited in the NCBI Sequence Read Archive (SRA) under accession numbers SRR22888886 and SRR22890161. Genome assemblies are available through NCBI BioProject numbers PRJNA911605 and PRJNA912836.
